# Retention outcomes and drivers of loss among HIV-exposed and infected infants in Uganda: a retrospective cohort study

**DOI:** 10.1186/s12879-018-3275-6

**Published:** 2018-08-22

**Authors:** Charles Kiyaga, Vijay Narayan, Ian McConnell, Peter Elyanu, Linda Nabitaka Kisaakye, Adeodata Kekitiinwa, Matthew Price, Jeff Grosz

**Affiliations:** 1 Ministry of Health AIDS Control Programme, Kampala, Uganda; 2 Clinton Health Access Initiative, Kampala, Uganda; 3 Baylor College of Medicine, Kampala, Uganda

**Keywords:** Uganda, HIV, HIV-exposed infant, Early infant diagnosis, Pediatric HIV, Prevention of mother-to-child transmission, Testing, Retention, Linkage to care

## Abstract

**Background:**

Uganda’s HIV Early Infant Diagnosis (EID) program rapidly scaled up testing of HIV-exposed infants (HEI) in its early years. However, little was known about retention outcomes of HEI after testing. Provision of transport refunds to HEI caregivers was piloted at 3 hospitals to improve retention. This study was conducted to quantify retention outcomes of tested HEI, identify factors driving loss-to-follow-up, and assess the effect of transport refunds on HEI retention.

**Methods:**

This mixed-methods study included 7 health facilities— retrospective cohort review at 3 hospitals and qualitative assessment at all facilities. The cohort comprised all HEI tested from September-2007 to February-2009. Retention data was collected manually at each hospital. Qualitative methods included health worker interviews and structured clinic observation. Qualitative data was synthesized, analyzed and triangulated to identify factors driving HEI loss-to-follow-up.

**Results:**

The cohort included 1268 HEI, with 244 testing HIV-positive. Only 57% (718/1268) of tested HEI received results. The transport refund pilot increased the percent of HEI caregivers receiving test results from 54% (*n* = 763) to 58% (*n* = 505) (*p* = .08). HEI were tested at late ages (Mean = 7.0 months, *n* = 1268). Many HEI weren’t tested at all: at 1 hospital, only 18% (67/367) of HIV+ pregnant women brought their HEI for testing after birth. Among HIV+ infants, only 40% (98/244) received results and enrolled at an ART Clinic. Of enrolled HIV+ infants, only 43% (57/98) were still active in chronic care. 36% (27/75) of eligible HIV+ infants started ART. Our analysis identified 6 categories of factors driving HEI loss-to-follow-up: fragmentation of EID services across several clinics, with most poorly equipped for HEI care/follow-up; poor referral mechanisms and data management systems; inconsistent clinical care; substandard counseling; poor health worker knowledge of EID; long sample-result turnaround times.

**Discussion:**

The poor outcomes for HEI and HIV+ infants have highlighted an urgent need to improve retention and linkage to care. To address the identified gaps, Uganda’s Ministry of Health and the Clinton Health Access Initiative developed a new implementation model, shifting EID from a lab-based diagnostic service to an integrated clinic-based chronic care model. This model was piloted at 21 facilities. An evaluation is needed.

## Background

Globally the number of infants newly infected with HIV has declined in recent years, from 370,000 in 2009 to 150,000 in 2015 [1, 2]. However, despite increasing access to anti-retroviral therapy (ART) in sub-Saharan Africa, diagnosis and treatment of HIV-positive (HIV+) infants has lagged behind [3, 4]. In Uganda, an estimated 91,000 HIV-exposed infants (HEI)— infants aged 0–18 months born to HIV+ mothers— were born in 2010, yet only 41,340 (46%) were tested [5, 6]. Out of 78,000 total HIV+ children (aged 0–14 years) who were eligible for ART in Uganda in 2010, only 24,031 (31%) were diagnosed and started on ART [6].

HIV+ infants must be diagnosed and treated early to survive. Unlike in adults, disease progression is rapid in HIV+ infants [7]. For HIV+ infants not initiated on ART, 35% are likely to die within the first year of life and 52% by 2 years of age [8]. Early initiation of HIV+ infants on ART slows disease progression, suppresses viral load, and dramatically reduces mortality rates [9–11]. Diagnosis of HEI is conducted in resource-limited settings by DNA Polymerase Chain Reaction (PCR) testing of Dried Blood Spot (DBS) samples [12, 13]. In Uganda, DBS samples are drawn from HEI at health facilities and referred to a specialized reference laboratory for DNA PCR testing.

**Fig. 1:**
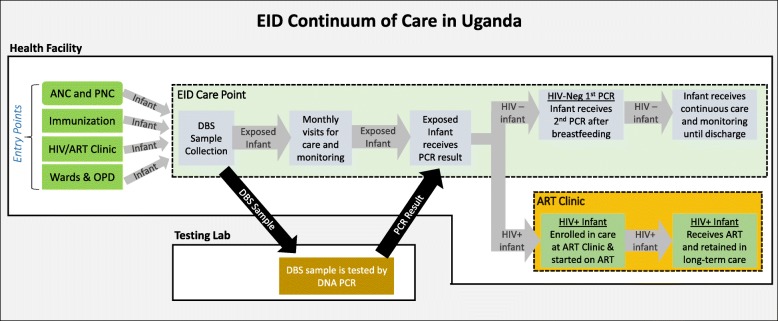
EID Continuum of Care in Uganda. *Diagram showing steps in the HIV infant diagnosis continuum:* HEI encounter the health facility at many ‘entry point’ clinics. Health workers (HWs) at entry points must proactively screen for HIV exposure and link the identified HEI to the facility’s testing point for DBS sample collection. The DBS sample must be dispatched to the reference lab, tested by DNA PCR, and result returned to the health facility by the time the HEI caregiver returns for their next appointment. If the PCR result is negative, the HEI must receive a 2nd confirmatory PCR test 6 weeks after cessation of breastfeeding. If the infant tests HIV+, the infant must be immediately enrolled at the HIV/ART Clinic and initiated on ART. ANC = Antenatal Clinic; PNC=Postnatal Clinic; ART = Antiretroviral Therapy; OPD = Outpatient Department; DBS = Dried Blood Spot; PCR = Polymerase Chain Reaction

As shown in Fig. 1, Early Infant Diagnosis (EID) is a complex process that has many points where HEI can be lost [14]. Since the diagnostic process for HEI can take several months, it is vital that all HEI receive regular clinical care (growth and developmental monitoring, clinical assessment) and Cotrimoxazole prophylaxis until their HIV status is confirmed. An HIV+ infant may become symptomatic from an infection before PCR results return. The onset of HIV clinical symptoms suggests advanced disease progression, and delaying treatment of symptomatic HIV+ infants increases risk of death [15, 16]. Therefore all HEI should be visiting the clinic regularly so that any opportunistic and other HIV-associated infections can be immediately recognized and treated; the infant can then be immediately diagnosed based on clinical status and initiated on ART. Cotrimoxazole prophylaxis is critical; it has been shown to reduce mortality by 43% for HIV+ infants who have not yet started ART [17]. Caregivers of HEI require intensive and continuous counseling about the testing process and proper feeding practices [18].

In Uganda, after successfully getting EID systems up and running, the Ministry of Health (MOH) initially focused on increasing the number of HEI tested and decentralizing EID services to a greater number of health facilities. PCR tests increased from 6437 in 2007 to over 37,000 in 2009 [5, 6]. However, little attention was paid to HEI outcomes after testing. No data was available at national level on whether HEI received test results and completed the testing algorithm, and whether HIV+ infants were enrolled at an HIV/ART Clinic, initiated on ART and active in care.

To improve retention of tested HEI, Uganda’s MOH and the Clinton Health Access Initiative (CHAI) piloted a transport refund program at three regional referral hospitals in mid-2008. Caregivers of HEI were provided transport reimbursement at each visit to encourage and enable them to return to the health facility to receive PCR test results and follow-up care. The cash amounts varied depending on how far the mother traveled.

The MOH and CHAI conducted this study at 7 health facilities to achieve three objectives: quantify the retention outcomes of tested HEI at 3 hospitals, identify the factors driving HEI loss to follow-up at each point in the EID continuum of care, and assess the effect of the transport refund pilot on HEI retention.

## Methods

This mixed-methods study was conducted at 7 government health facilities in Uganda— retrospective cohort review of tested HEI at 3 regional referral hospitals, and qualitative assessment at all 7 health facilities. The selected facilities cut across different health facility levels and geographic regions (Table 1).

**Table 1:** Health facilities in this study

Facility Name	Facility Level	Region	Cohort Review?	Qualitative Assessment?
Masaka Hospital	Regional Referral Hospital	Southwest	Yes	Yes
Jinja Hospital	Regional Referral Hospital	East	Yes	Yes
Lira Hospital	Regional Referral Hospital	North	Yes	Yes
Kayunga Hospital	District Hospital	East-Central	No	Yes
Wakiso H/C IV	Health Center IV	Central	No	Yes
Mukono H/C IV	Health Center IV	East-Central	No	Yes
Namayumba H/C IV	Health Center IV	Central	No	Yes

The five levels of the health system are 1) Regional Referral Hospital, 2) District Hospital, 3) Health Center IV, 4) Health Center III, and 5) Health Center II. During the review period, EID and pediatric ART services were mostly provided at the level of ‘Health Center IV’ and above

The retrospective patient cohort review tracked retention outcomes of all HEI aged 6 weeks to 18 months tested by DNA PCR between September 2007 and February 2009 (*n* = 1268 HEI). To identify the facility-level factors driving loss-to-follow-up of HEI, qualitative assessment of EID program implementation was done using 2 methods: semi-structured interviews with HWs and structured observation of health facility systems. Ethical approval for this study was obtained from the Mildmay Uganda Research and Ethics Committee.

### Data collection and analysis for cohort review

All DNA PCR tests of HEI in Uganda were captured in a national electronic database housed at the MOH EID office. After a sample was tested by DNA PCR at the regional reference lab, a copy of the result was sent to the MOH EID office and captured in a Microsoft Excel database. At the outset of this study, a list of HEI who received a DBS test at the 3 hospitals during the review period was extracted into a separate Excel database for this study. The extract from the national EID database provided the date of DBS test, age at DBS test, and HIV test result for each HEI in the cohort.

The retention data for each HEI was then manually collected at each hospital from 10+ data sources spread across several different clinics. Data was collected on every follow-up visit made by each HEI in the cohort and all other variables of interest (Table 2) and inputted into the study database. In addition to the HEI cohort review, we collected data at 1 hospital on the number of HIV+ pregnant women whose infants were tested after birth, which provided a measure of HEI testing coverage.

**Table 2:** Key variables of interest and data sources at the hospital

Variables	Data Sources	Clinics within Health Facility
• Dates of each HEI follow-up visit	• DBS Dispatch Register	• Lab
• Purpose of visits	• EID Follow-Up Cards	• ART Clinic
• Whether test results received	• EID Data Register	• Antenatal Clinic
• When test results received	• Transport Refund Register	• Postnatal Clinic
• Breastfeeding status	• Test result slips for HEI	• Immunization Clinic
• Provision of Cotrimoxazole prophylaxis	• Homemade clinic attendance registers	• Outpatient Department
• Clinical care indicators	• Homemade HEI clinic attendance registers	• Feeding Units
• When due for 2nd PCR	• Homemade follow-up register for HIV-exposed infants	• Pediatric Ward
• Whether transport refund given	• Antenatal, Postnatal & Immunization Registers	• Maternity Ward
• Dates of sample dispatch and result return to facility	• Pediatric Ward Register	• Adult Wards
• Referring clinic (entry point for HEI)	• Outpatient Register	
• HIV+: If/when enrolled at ART clinic	• Pre-ART & ART Registers	
• HIV+: Dates of each visit at ART clinic	• Patient files for HIV+ patients	
• HIV+: If initiated on ART and when		
• HIV+: CD4 tests and clinical stage		

*HEI* HIV-exposed infant, *PCR* Polymerase Chain Reaction, *ART* Antiretroviral Therapy, *DBS* Dried Blood Spot, *EID* Early Infant Diagnosis

The data was analyzed using Excel and SPSS software. 95% confidence intervals were calculated for mean values of age at testing, enrollment into care, and ART initiation. Statistical significance of retention changes before and after the transport refund intervention was assessed using a z-test for comparison of proportions in two independent samples (α = .05).

### Qualitative assessment: Data collection, analysis, and triangulation

The qualitative assessment of EID implementation was designed to identify the factors contributing to HEI loss-to-follow-up. Study investigators conducted semi-structured interviews with 37 HWs across the 7 facilities to elicit information on practices and systems in EID program implementation, as well as capture their experiences and perspectives as direct implementers. Investigators conducted structured non-participant observation of EID implementation systems at the different clinics involved in EID within each facility (e.g. entry point clinics, lab, HIV clinic). Whereas the observations provided direct examination of EID practices and systems (e.g. flow mapping), the interviews provided information and context through the lens of the HW.

The interviews and observations captured how, when, where and by whom EID services were provided at the health facility; the flow of caregivers, samples, test results, and data; the data management and referral tools in use; and the package of care and counseling services provided to HEI. Topical guides were used for the interviews and observations, and the data was recorded in field note format. For each method, the raw qualitative data was typed into Microsoft Word, collated by topic and question, indexed, and analyzed to identify common themes. The analyses from direct observation data and interview data were triangulated [19]; we first generated broader categories of factors affecting testing and retention of HEI, then identified specific factors within each category, and finally broke down the factors by which part(s) of the EID continuum they affected.

The interviews also assessed HW knowledge and awareness of EID and pediatric ART (how to screen for HIV exposure, signs of pediatric HIV infection, testing and feeding algorithms, infant ART initiation criteria), as well as the counseling messages conveyed to caregivers. To obtain a basic measure of HW knowledge, we calculated how many of the interviewed HWs provided accurate information about each topical area.

We used both interviews and observations to assess whether and how consistently clinical care and counseling were provided to HEI. Given the lack of clinical care and counseling data for individual HEI visits, our assessment focused more broadly on whether each key component of HEI clinical care and counseling package was provided as 1) routine practice at every visit, with few exceptions, 2) provided occasionally or only when triggered for a specific reason, or 3) not provided at all, except in rare situations. To assess the messaging provided by HWs to caregivers during counseling (as a proxy measure for counseling ‘quality’), we compared the information conveyed by HWs with the counseling messages prescribed in the national EID training manual [20].

## Results

### Outcomes for HIV-exposed and HIV-positive infants

This study tracked retention outcomes of 1268 HEI who received a DNA PCR test at 3 regional referral hospitals in Uganda between September 2007 and February 2009. 244 out of 1268 HIV-exposed infants tested HIV-positive (19.2%).

#### Identification and testing of HEI

The number of HEI in the catchment area of the three hospitals was unknown. However, the number of HIV+ pregnant women receiving Prevention of Mother-to-Child Transmission (PMTCT) services at the hospitals served as a reasonable proxy for the minimum number of HEI needing to be tested. At one hospital, only 18% (67/367) of HIV+ pregnant women brought their infants back for DBS testing after birth. In addition, analysis of qualitative observation and interview data revealed that HWs at many other entry points (e.g. outpatient department, immunization, feeding units, wards) were not proactively screening infants for possible exposure to HIV, nor taking active steps to ensure that identified HEI successfully reached the EID testing point.

#### Age at testing for HEI

The mean age of first DBS test was 7.0 months of age among the 1268 HEI who were tested at the 3 hospitals (SD = 5.9). (Table 3) Only 24.1% were tested at ≤2 months of age. Among infants testing HIV+, the mean age of first DBS test was 9.6 months (*n* = 244, SD = 7.3), mean age of enrolment into care was 13.9 months (*n* = 86, SD = 10.4) and mean age of ART initiation was 16.1 months (*n* = 27, SD = 11.9).

**Table 3:** Age at testing, enrollment and ART initiation for HIV-exposed and infected infants

All HIV-Exposed Infants	Mean Age in Months (95% CI), SD	N
Age at 1st DBS Test	7.0 (6.7–7.3), SD = 5.9	1268
HIV+ Infants Only		
Age at 1st DBS Test	9.6 (8.7–10.5), SD = 7.3	244
Age at enrollment in ART Clinic	13.9 (11.7–16.0), SD = 10.4	86
Age at ART initiation	16.1 (11.6–20.6), SD = 11.9	27

*DBS* Dried Blood Spot, *ART* Antiretroviral Therapy, *CI* Confidence Interval, *SD* Standard Deviation

#### HEI receiving test results and completing testing algorithm

Only 57% of the 1268 HEI received their PCR test results. Out of the 261 HEI who had a negative 1st PCR at less than 6 months of age and received their results, only 18.4% had a confirmatory 2nd PCR test. Breastfeeding status of HEI was not documented making it difficult to know the exact number requiring a confirmatory PCR test, but according to national estimates, greater than 90% of HIV+ mothers in Uganda breastfed during the review period [6, 21].

#### Effect of transport refund pilot on HEI receiving test results

**Fig. 2:**
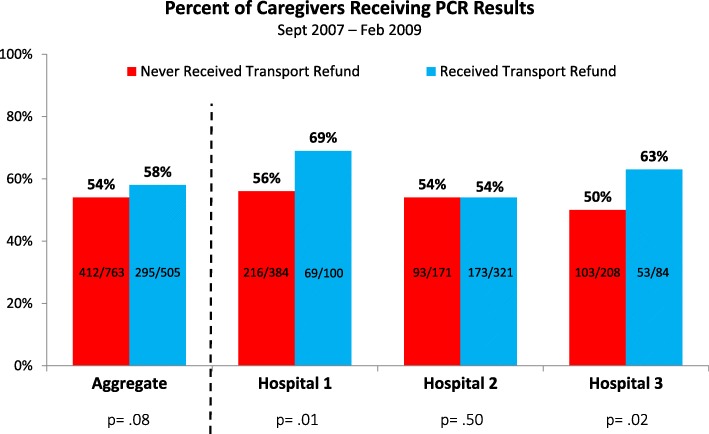
Effect of Transport Refund Pilot on Retention of HIV-Exposed Infants. Graph showing the percent of caregivers receiving results, among those who received the transport reimbursement intervention versus those who did not receive it. PCR = Polymerase Chain Reaction

Figure 2 shows the percent of caregivers receiving PCR results among those receiving at least one transport refund (post-intervention period of May 2008 – February 2009) and those never receiving a refund (pre-intervention period of September 2007–April 2008). For the three hospitals in aggregate, retention increased from 54% (*n* = 763) to 58% (*n* = 505) (*p* = .08). The individual hospitals had increases of 13% (*p* = .01), 0% (*p* = .50) and 13% (*p* = .02). Even when considering only the 2 hospitals with a statistically significant gain in retention, the percent receiving results was still under 70% for both. The average cost of the program per HEI was 21 USD (average of 7 USD per visit, and 3 visits per HEI).

#### Outcomes for HIV+ infants

**Fig. 3:**
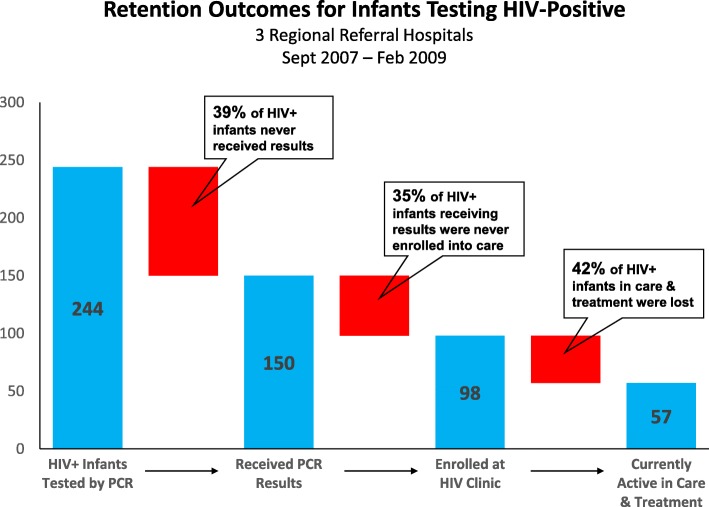
Retention Outcomes for Infants Testing HIV-Positive. Graph showing the outcomes for HIV-exposed infants testing HIV-positive by DNA PCR. The red bars represent the number of infants lost-to-follow up at each step in the EID process. PCR = Polymerase Chain Reaction

Figure 3 shows the outcomes for the 244 infants testing HIV+ at the 3 hospitals. Out of 244 infants testing HIV+, only 150 returned to the health facility and received results (39% loss). Out of the 150 HIV+ infants receiving results, 98 enrolled into care at the HIV/ART Clinic (35% loss). Finally, 57 of the 98 who enrolled at the HIV/ART Clinic were still alive and active in care as of January 2010 (42% loss). Overall, only 23.4% of HIV+ infants diagnosed through the EID program were still active in care. 75 of the 98 HIV+ infants enrolled in care at the HIV/ART Clinic were eligible for ART under national guidelines during the review period, but only 36% (27/75) were initiated on ART.

### Drivers of HEI loss throughout the EID continuum of care

Synthesis, analysis and triangulation of qualitative data from the HW interviews and direct clinic observations yielded 6 categories of factors contributing to HEI loss, as well as the specific factors within each category and which part(s) of the EID continuum they affected (Table 4). The 6 categories included EID clinic systems and patient flow; referral and data management systems; clinical care and visit schedule; counseling materials, messages and techniques; knowledge and awareness of health workers, and DBS sample-result turnaround time.

**Table 4:** Drivers of Loss at Each Point in the EID Continuum of Care

	*HEI not identified and tested*	*HEI not receiving results and completing testing algorithm*	*HIV+ infants not enrolling in care at HIV/ART Clinic*	*HIV+ infants not started on ART & retained in chronic care*
EID Clinic Systems and Patient Flow	• EID fragmented across multiple clinics that are poorly equipped to test, follow-up and provide care for HEI • Ad-hoc and ineffective flow of caregivers, samples, results, and data within the health facility			
Referral and Data Management Systems	• No referral mechanisms to link HEI from entry points to EID testing	• No longitudinal tracking tools or appointment systems for HEI	• No referral mechanisms to link HIV+ infants from EID to HIV/ART Clinic	
Clinical Care and Visit Schedule	• Poor clinical care and HEI not visiting every month: prevents early identification of opportunistic and other HIV-associated infections • Clinical care not integrated into EID services: undermines importance of HEI returning regularly to clinic			
Counseling Messages, Materials and Techniques	• Caregivers not convinced to get their HEI tested	• Caregivers not convinced to return to the clinic	• Caregivers not convinced to enroll HIV+ infant	• Caregivers hesitant to start & adhere to ART
Knowledge and Awareness of Health Workers	• Many HWs do not know how to screen and identify HEI	• Many HWs not proficient in HEI testing and feeding algorithm	• Many HWs not proficient in testing/feeding algorithm	• Many HWs do not know ART criteria; uncomfortable initiating infants on ART
DBS Sample-Result Turnaround Time		• Test results not back in time for caregiver visits		

*HEI* HIV-Exposed Infant, *HW* Health Worker, DBS: Dried Blood Spot ART: Antiretroviral Therapy

#### Drivers of loss: EID clinic systems

**Fig. 4:**
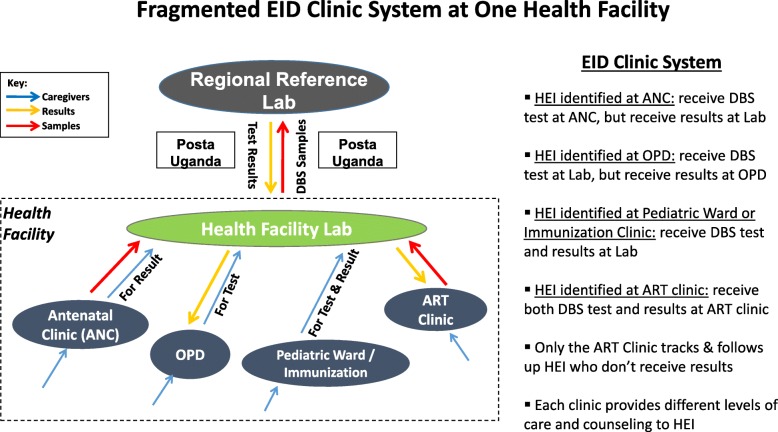
Fragmented EID Clinic System at One Health Facility. Diagram depicting the flow of caregivers, samples, and results at one of the reviewed health facilities. ANC = Antenatal Clinic, DBS = Dried Blood Spot, OPD = Outpatient Department, HEI=HIV-Exposed Infant

An example of the clinic system from a reviewed facility is shown in Fig. 4. Most facilities had an ad hoc and fragmented EID clinic system— particularly the flow of caregivers, samples, and test results— that contributed to HEI getting lost throughout the EID continuum. Health facilities did not have one, clear, well-equipped place where all HEI caregivers would go for registration and post-test counseling on the day of the DBS test, and then return to receive results and follow-up care. Instead, at some facilities DBS testing was decentralized to entry point clinics, but all results were given from the main lab. At other facilities, all HEI were referred to the main lab for the DBS test, but result distribution was decentralized to the entry point where the HEI was identified. Most often, however, it was a combination of the two systems, with HEI receiving a DBS test and results at different places within the same facility depending on the entry point clinic where they were identified; consequently, HEI caregivers and HWs often didn’t know where PCR results were given. Data management, follow-up, care provision and counseling of HEI were fragmented across different clinics.

**Fig. 5:**
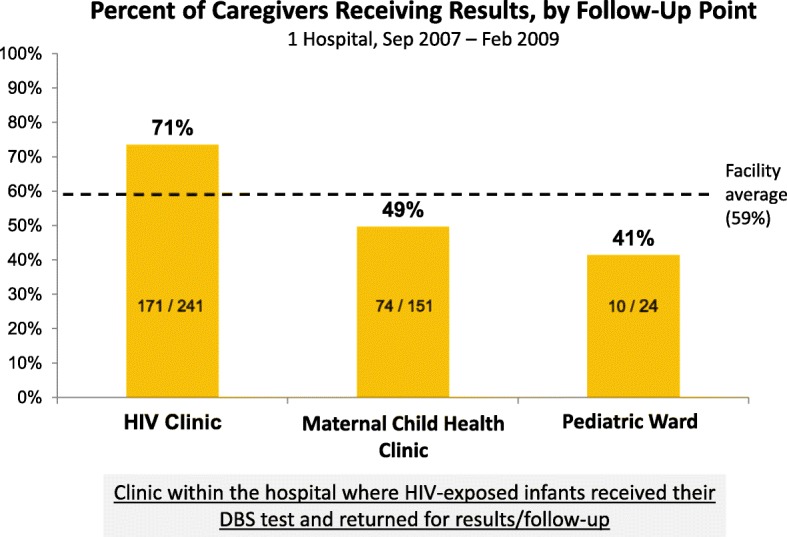
Relationship between "location of HIV-exposed infant follow-up" and "percent of caregivers receiving PCR results". Graph showing the percent of caregivers receiving results at 1 hospital, by testing/follow-up point

At one hospital, test results were returned to caregivers at three different places depending on where HEI initially received their DBS test (Fig. 5): 71% of the HEI tested and followed up at the ART Clinic received results (*n* = 241), compared to 49% of HEI tested and followed up at the Maternal-Child Health Clinic (*n* = 151) and 41% of HEI tested and followed up at the pediatric ward (*n* = 24) (*p* < .0001 for comparison of ART Clinic versus other points). Interviewed HWs at all three units of the hospital expressed that the ART clinic was the best location for providing PCR results and all follow-up care; they noted that because HIV+ mothers were already attending the ART Clinic monthly for their own chronic care, having HEI follow-up visits there allowed both mother and baby to receive care simultaneously, making it more likely that the infant would receive regular care and PCR results.

At several reviewed facilities, HEI caregivers received results at the facility’s laboratory. Several HWs noted that the lab was a centralized place for giving results and often most convenient for the facility, but that it was a poor location for result delivery because laboratory technicians in Uganda are not professionally qualified to provide sensitive post-test counseling to HEI caregivers, and also because the lab cannot not provide care or follow-up of HEI.

#### Drivers of loss: Referral systems and data management tools

Lack of mechanisms for referral within the health facility contributed to HEI loss at multiple points in the EID continuum of care: linkage of HEI from entry points to testing point, and linkage of HIV+ infants from EID services to the HIV/ART Clinic. The earliest point of HEI identification is when the HIV+ mother is still pregnant and receiving PMTCT services in the facility’s antenatal clinic. However, there was no system in place for the antenatal clinic to provide documentation to the EID testing point, preventing the EID testing point from knowing that there would be an HEI needing a DBS test after delivery and in what month, and following up in the community if the HEI didn’t come. Similarly, when HEI were identified at entry points such as the outpatient department and immunization clinic, caregivers were told verbally to attend the EID testing point but no information was given to the EID testing point. Likewise, after HIV+ infants received their PCR results, the ART Clinic was not informed that an HIV+ infant needed to be enrolled and did not have any contact information.

Poor data management systems and tracking tools contributed to HEI not receiving results and failing to complete the testing algorithm. There was no system to document and manage appointments for clinic visits, which prevented HWs from identifying the HEI who did not return to the health facility and following them up by phone or home visit. The data register for HEI captured only the first visit when the DBS test was done. Because the register did not capture any follow-up visits, HWs didn’t know where HEI were in the testing algorithm— e.g. whether they had received 1st PCR results, whether they were still breastfeeding, and when they were due for a 2nd PCR test.

#### Drivers of loss: Clinical care, prophylaxis, and counseling

Among the 7 reviewed facilities, clinical care wasn’t provided to HEI as routine practice, and barely at all in many cases (Table 5). Only 3/7 facilities provided Cotrimoxazole as routine practice at every HEI visit, only 3/7 measured weight regularly, none measured height or monitored developmental milestones. 5/7 facilities regularly conducted a clinical assessment for HIV signs and symptoms. Clinical care was not integrated into EID services and there was no standard HEI visit schedule— caregivers visited the clinic only for the purpose of receiving DBS tests or results. EID wasn’t viewed by HWs and caregivers alike as a chronic care service in which routine visits were essential for HEI survival.

**Table 5:** Care and Counseling Provided to HIV-Exposed Infants at 7 Health Facilities

	Regional Referral Hospital	Regional Referral Hospital	Regional Referral Hospital	District Hospital	Health Center IV	Health Center IV	Health Center IV
Cotrimoxazole prophylaxis	✓●	**✓✓**	**✓✓**	**✓✓**	–	–	✓●
Growth monitoring- weight	–	**✓✓**	✓●	**✓✓**	–	–	–
Growth monitoring- height	–	–	–	–	–	–	–
Immunization assessment and referral	–	✓●	–	**✓✓**	–	–	–
Clinical assessment for signs/symptoms of HIV	**✓**●	**✓**●	**✓**●	**✓**●	**✓**●	–	–
Developmental assessment	–	–	–	–	–	–	–
Counseling on nutrition and feeding practices	**✓**●	**✓**●	**✓**●	**✓**●	**✓**●	–	–
Counseling on testing algorithm and EID process	**✓**●	**✓**●	**✓**●	**✓**●	**✓**●	–	–
KEY: **✓✓ Always provided:** provided as routine practice at every clinic visit, with rare exceptions **✓**● **Sometimes provided:** provided occasionally, ad hoc, or only when triggered for specific reason(s) **— Rarely or never provided:** not provided at all except in rare situations							

Assessment of clinical care provided to HIV-exposed infants and counseling of their caregivers. Results were derived from analysis and triangulation of qualitative data from direct observation of HIV-exposed infant clinic visits and interviews with health workers

Also shown in Table 5, counseling was inconsistently provided to HEI caregivers on the testing process, feeding, and nutrition— during pregnancy, after birth at the identification/referral point, and at the EID testing point. In addition to inconsistency of counseling provision, quality was an issue. Analysis of data from observation of counseling sessions and HW interviews revealed that HWs were not conveying many of the key messages needed to adequately educate mothers about why they needed to get their babies tested, the diagnostic process, and steps to keep their baby alive and healthy.

HWs reported that they had not been adequately trained on techniques to effectively convey the key information, noting the particular challenges of counseling in Uganda’s HIV context. These gaps were compounded by the absence of counseling reference materials for HWs and informational materials for HEI caregivers; some HWs pointed out that similar materials were effective in other non-HIV programs.

#### Drivers of loss: Knowledge and awareness of health workers

Many HWs lacked sufficient knowledge and awareness about EID and pediatric HIV. We found that HWs weren’t proactively checking exposure status and actively identifying HEI at many entry points, stemming from insufficient knowledge (27/37 weren’t proficient with the process for screening infants of HIV+ mothers for HIV exposure) and poor participation (interviewed HWs did not always perceive proactive screening as one of their core job functions). 30/37 HWs could not list more than two common clinical signs of pediatric HIV infection, and HWs at entry points acknowledged that they may have failed to recognize clinical presentations as opportunistic or other HIV-associated infections. 29/37 interviewed HWs didn’t know the full infant HIV testing algorithm and proper feeding guidelines, likely contributing to substandard counseling of caregivers.

We found that many interviewed HWs (29/37) didn’t know the correct ART initiation criteria. Further, several clinical staff working in ART clinics reported that they didn’t want to initiate children on ART because they weren’t comfortable with the complicated treatment regimens for HIV+ children, noting that they were afraid to make fatal mistakes and needed more hands-on training to first build their confidence.

Most HWs reported not having been trained in EID recently or at all. Among those who were trained, several noted that the one-day EID trainings at the facility were not sufficient for them to retain the information and master the skills they were newly introduced to. HWs complained that there were no organized follow-up trainings and mentorships, and also noted the high rates of HW transfer and turnover as challenges. HWs also pointed out that the training did not include all of the topics discussed in our interviews, and some reported that they were never oriented on the most recently updated EID guidelines.

#### Drivers of loss: Sample-result turnaround time

**Fig. 6:**
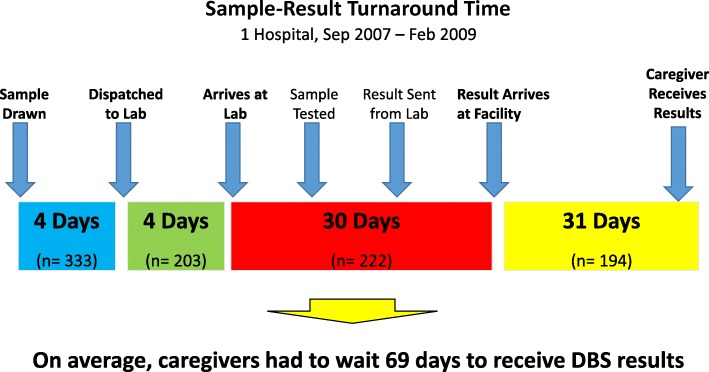
Sample-result turnaround time at one hospital. Diagram shows the average number of days it took for each step in the sample referral process. The n values differ for each point in the sample referral process because health workers inconsistently documented dates for ‘sample draw’, ‘dispatched to lab’ and ‘arrives at lab’ in facility and lab data registers. The n values for ‘result arrives at facility’ and ‘caregiver receives results’ are different because not every tested infant received PCR results

**Fig. 7:**
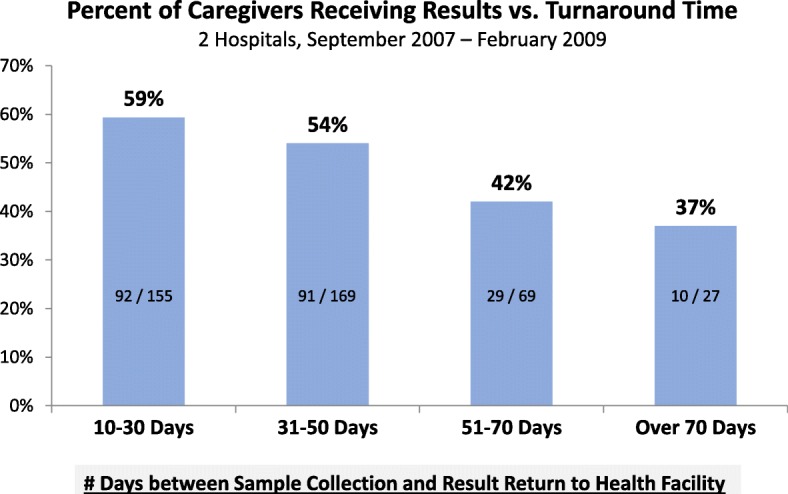
Graph shows the percent of caregivers receiving PCR results as a function of sample-result turnaround time at 2 hospitals. The caregiver was due to return for test results 1 month after the DBS test, so the desired turnaround time for the EID program was in the range of ‘10–30 days’

Long sample-result turnaround time (TAT) also contributed to caregivers not receiving results— interviewed HWs expressed frustration that caregivers would come back 1 month after the DBS test only to find out that the result had not yet returned from the reference lab, and asserted that caregivers were disinclined to come back again because of this. Figure 6 shows TAT data from one of the hospitals. Test results took an average of 38 days to get back to the facility after the DBS sample was drawn (*n* = 222), and an additional 31 days to be given to the caregiver (among the subset of caregivers who received results, *n* = 194). Data from 2 of the hospitals shows that with larger sample-result TATs, fewer caregivers received results Fig. 7. However, even when PCR results came back within the desired range of 10–30 days, less than 60% of caregivers ultimately received them (*n* = 155). Data from another hospital showed that among caregivers who didn’t receive results, 73% did not make even a single follow-up visit (*n* = 201); TAT duration had no effect on this group of lost HEI.

## Discussion

This study is the first-ever measure of longitudinal outcomes of HEI in Uganda. The findings are striking: only 40% of infants testing HIV+ ultimately enrolled at an ART clinic, and only 18% of all HIV+ infants were ultimately alive and retained in care. Only 57% of tested HEI received their 1st PCR test results. Among HEI with a negative 1st PCR, very few had a 2nd PCR, suggesting that transmission through breastfeeding was not being diagnosed. Mean age of testing was 7.0 months among all HEI, and 9.6 months among HIV+ infants, suggesting that many infected infants were captured only after they had already become sick. By such late ages, the disease may have progressed beyond the point of no return. Many HEI were not tested at all: only 18% of HIV+ pregnant women brought their HEI back for testing at 1 hospital, suggesting a major gap in linkage between PMTCT and EID services.

Lack of routine specialized care provision for HEI contributed to attrition of HIV+ infants throughout the EID continuum of care. While PCR results were pending, the infants who were HIV+ may have become symptomatic and required immediate ART. Since HEI were not coming to the clinic regularly for care check-ups and medication refills, clinical indicators of HIV infection may have been missed (e.g. weight loss, stunting); opportunistic or other HIV-associated infections may have gone untreated until it was too late. The absence of routine care undermined the importance of HEI returning to the facility regularly. If caregivers had been visiting the clinic monthly for care and counseling, it is likely they would have eventually received results at one of the visits and completed the testing algorithm, even when PCR results were delayed in getting back to the facility.

### Implications for Uganda’s EID program and next steps

**Fig. 8:**
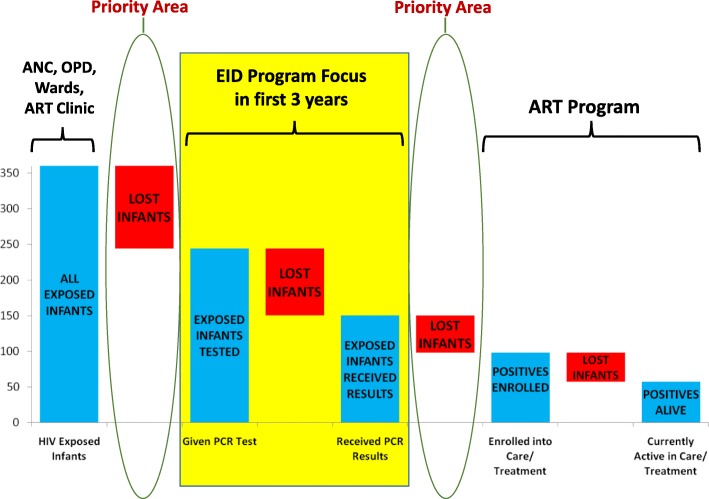
Diagram depicting areas of the EID continuum of care where the national program focused in its initial years (testing and caregiver retrieval of results), and highlighting priority areas for improvement (linkage of HEI from entry points to testing, and linkage of HIV+ infants from result retrieval point to HIV clinic). ANC = Antenatal Clinic, OPD = Outpatient Department

Uganda’s national program has succeeded in rapidly scaling up testing of HEI and has started to make inroads in improving retention of HEI after testing. In addition to intensifying retention efforts, the program’s focus should now expand to include linkage of HEI from entry points to DBS testing, and linkage of HIV+ infants to chronic care after receiving results Fig. 8. The study findings also underscore the need to improve EID’s integration with PMTCT and pediatric ART services at national and district levels.

Findings from analysis of the transport refund pilot can help inform national program strategy. While more HEI caregivers received test results at 2 of the 3 hospitals among those who received the transport reimbursements, the overall improvement was modest and retention was still under 70% in the best case. This suggests that transport costs for caregivers, while one contributing factor, were not the only or biggest driver of HEI caregivers failing to receive results. Further, the high cost of implementing the program suggests challenges with sustainability and scalability of this type of intervention, even if the retention gains had been greater. Ultimately, addressing the underlying barriers to HEI retention by improving health facility systems for how EID is implemented may provide a more feasible, cost-effective, and long-term solution.

Efforts to improve retention of tested HEI in Uganda have emphasized reduction of sample-result TAT. Indeed, the national EID program has undertaken several initiatives to reduce TAT, including creation of a new sample-result transport system and consolidation of the regional reference laboratories into a single, more efficient centralized national laboratory [22–24]. Our study found that sample-result TAT was too long and contributed to loss to follow-up, but even when TAT was less than 30 days, far too few caregivers received results (< 60%). Also, lower TAT wouldn’t have a difference for the many lost HEI who never returned after the test. The key takeaway is that improving sample-result TAT needs to be one key component of a multi-faceted retention strategy, rather than the primary focus.

**Fig. 9:**
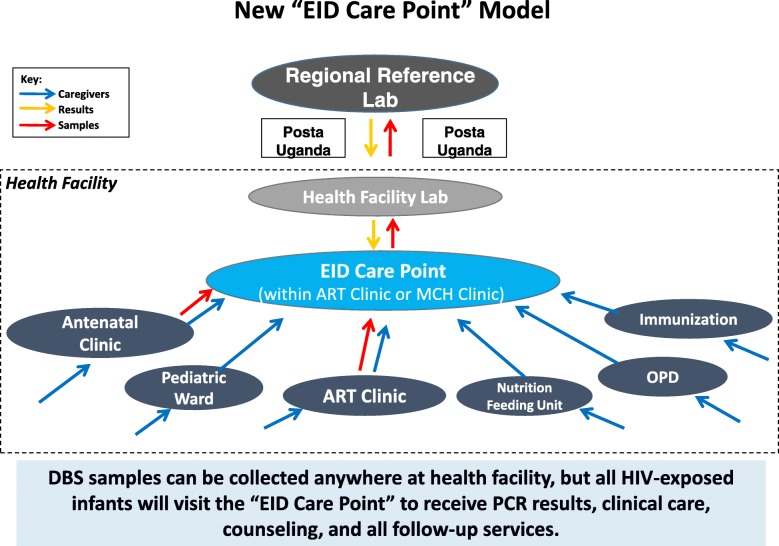
New “EID Care Point” Model. Diagram depicting the new proposed model for implementation of EID services at health facilities in Uganda. DBS = Dried Blood Spot, PCR = Polymerase Chain Reaction, EID = Early Infant Diagnosis

On a fundamental level, the EID program at facility-level needs to shift from a lab-based service to a clinic-based chronic care service. Based on this study’s findings, we have developed a new model for EID implementation at health facilities, depicted in Fig. 9. In this model, HEI receive the full complement of EID services—clinical care, counseling, registration and follow-up— at one centralized and well-equipped “EID care point” in the health facility. This “EID care point” sits within an existing clinic— ideally in the HIV/ART Clinic— and contains sufficient space, basic medical equipment, at least one dedicated clinical staff, data tools, and job aides to enable high-quality care, tracking, follow-up, and linkage of HEI. In contrast to the fragmented systems previously in place, all HEI are initially referred to the “EID care point” for registration and counseling, and return there for PCR results and all follow-up care visits. DBS tests can be done at the entry point clinics, “EID care point”, or facility lab, but all patients, samples and results run through the “EID care point”. The facility lab is a conduit for samples and results rather than the primary EID service delivery point. Each health facility adopts an EID clinic flow that works best for its context, but fulfills the minimum requirements of the “EID care point” model. The logic of the “care point” model lies in the premise that it is more effective to concentrate limited staffing and material resources for chronic care, counseling, tracking, and follow-up of HEI in one place, rather than scattering the focus and resources across many points in the facility.

To comprehensively address the gaps at each point in the EID continuum of care, the “EID care point” model is coupled with many other elements: instituting a routine monthly visit schedule for all HEI; establishing clear HEI clinical management protocols and providing clinical charts; setting up a triplicate referral system; providing medical equipment (e.g. height boards), longitudinal data registers, appointment books, counseling brochures and reference materials, job aides on HEI care, testing, and feeding, and financial support for follow-up of lost HEI. This set of initiatives is implemented through training and frequent on-site mentorship of health workers.

## Conclusions

The study findings were disseminated within Uganda to relevant MOH departments, district health teams, health workers, NGO technical partners, donors, and international agencies (UN, WHO). Uganda’s MOH and CHAI subsequently piloted the study recommendations (the “EID Systems Strengthening” program) at 21 health facilities, and the model was ultimately scaled up. An evaluation of the program model is critically needed to assess its impact and inform future strategies to improve HEI retention in Uganda.

## Abbreviations

ART: Antiretroviral Therapy
CHAI: Clinton Health Access Initiative
DBS: Dried Blood Spot
DNA: Deoxyribonucleic Acid
EID: Early Infant Diagnosis
HEI: HIV-Exposed Infant(s)
HIV: Human Immunodeficiency Virus
HW: Health Worker
MOH: Ministry of Health
PCR: Polymerase Chain Reaction
PMTCT: Prevention of Mother-to-Child Transmission
TAT: Turnaround time
